# Habitual intake of flavonoid subclasses and risk of colorectal cancer in 2 large prospective cohorts^1^,^2^

**DOI:** 10.3945/ajcn.115.117507

**Published:** 2015-11-04

**Authors:** Katharina Nimptsch, Xuehong Zhang, Aedín Cassidy, Mingyang Song, Éilis J O’Reilly, Jennifer H Lin, Tobias Pischon, Eric B Rimm, Walter C Willett, Charles S Fuchs, Shuji Ogino, Andrew T Chan, Edward L Giovannucci, Kana Wu

**Affiliations:** 3 Molecular Epidemiology Research Group, Max Delbrück Center for Molecular Medicine, Berlin, Germany;; 4 Department of Nutrition and; 5 Department of Epidemiology, Harvard T.H. Chan School of Public Health, Boston, MA;; 6 Channing Division of Network Medicine, Department of Medicine and; 7 Department of Pathology, Brigham and Women's Hospital, and Harvard Medical School, Boston, MA;; 8 Department of Nutrition, Norwich Medical School, University of East Anglia, Norwich, United Kingdom;; 9 Safety Statistics and Observational Research Analytics, Quantitative Sciences, Takeda Development Center Americas Inc., Deerfield, IL;; 10 Department of Medical Oncology, Dana-Farber Cancer Institute and Harvard Medical School, Boston, MA; and; 11 Division of Gastroenterology, Massachusetts General Hospital, Boston, MA

**Keywords:** flavonoids, flavonols, flavones, flavanones, flavan-3-ols, anthocyanins, colorectal cancer

## Abstract

**Background:** Flavonoids inhibit the growth of colon cancer cells in vitro. In a secondary analysis of a randomized controlled trial, the Polyp Prevention Trial, a higher intake of one subclass, flavonols, was statistically significantly associated with a reduced risk of recurrent advanced adenoma. Most previous prospective studies on colorectal cancer evaluated only a limited number of flavonoid subclasses and intake ranges, yielding inconsistent results.

**Objective:** In this study, we examined whether higher habitual dietary intakes of flavonoid subclasses (flavonols, flavones, flavanones, flavan-3-ols, and anthocyanins) were associated with a lower risk of colorectal cancer.

**Design:** Using data from validated food-frequency questionnaires administered every 4 y and an updated flavonoid food composition database, we calculated flavonoid intakes for 42,478 male participants from the Health Professionals Follow-Up Study and for 76,364 female participants from the Nurses’ Health Study.

**Results:** During up to 26 y of follow-up, 2519 colorectal cancer cases (1061 in men, 1458 in women) were documented. Intakes of flavonoid subclasses were not associated with risk of colorectal cancer in either cohort. Pooled multivariable adjusted RRs (95% CIs) comparing the highest with the lowest quintiles were 1.04 (0.91, 1.18) for flavonols, 1.01 (0.89, 1.15) for flavones, 0.96 (0.84, 1.10) for flavanones, 1.07 (0.95, 1.21) for flavan-3-ols, and 0.98 (0.81, 1.19) for anthocyanins (all *P* values for heterogeneity by sex >0.19). In subsite analyses, flavonoid intake was also not associated with colon or rectal cancer risk.

**Conclusion:** Our findings do not support the hypothesis that a higher habitual intake of any flavonoid subclass decreases the risk of colorectal cancer.

See corresponding editorial on page 3.

## INTRODUCTION

Flavonoids are a group of bioactive polyphenolic compounds naturally occurring in plant-based foods such as fruits, vegetables, grains and herbs, and drinks such as tea, wine, and juices. Based on their structure, flavonoids can be subclassified as flavonols, flavones, flavanones, catechins (flavan-3-ols or flavanols), isoflavones, anthocyanins, and oligomeric or polymeric flavonoids. Experimental studies have shown cancer-protective effects of flavonoids through several biological mechanisms, including antioxidative and anti-inflammatory properties, induction of apoptosis, and inhibition of proliferation and angiogenesis (1). Specifically, flavonoids have been shown to inhibit growth of human colon cancer cell lines in vitro and restrict colorectal carcinogenesis in animal models (2, 3). A proportion of dietary flavonoids is absorbed in the small intestine and reaches plasma concentrations that could have biological effects. In addition, substantial amounts reach the colon, where they are further metabolized into metabolites that may also mediate some biological activity (4). Current observational studies investigating the association between flavonoid intake and risk of colorectal cancer (CRC)^12^ have been inconsistent. Although several case-control studies suggest an inverse association between high flavonoid intake, particularly flavonols and anthocyanins, and CRC (5–9), prospective cohort studies have generally failed to detect an association (10–14). However, an inverse association between catechin intake and risk of rectal cancer has been suggested by a cohort study in women (15). In addition, in a secondary analysis of a US-based randomized controlled trial, the Polyp Prevention Trial, higher intake of flavonols was statistically significantly associated with a 76% reduced risk (OR: 0.24; 95% CI: 0.11, 0.53) of advanced recurrent adenoma, a precursor for CRC (16). In the only study investigating biomarkers of flavonoid intake in relation to CRC incidence we are aware of, urinary catechins at baseline were inversely related to later risk of colon cancer (17).

Dietary flavonols and flavones and CRC risk have been prospectively investigated in the Health Professionals Follow-Up Study (HPFS) and Nurses’ Health Study (NHS) previously, and no association was observed (13). A drawback of most prospective studies, including the previous analysis of NHS and HPFS data, is that only a small number of flavonoid subclasses (e.g., flavonols and flavones) were evaluated and intake ranges were limited (10–13, 15). In the present study, we have updated our flavonoid food composition database based on an updated and expanded USDA database (18) to examine the association between habitual intake of various flavonoid subclasses (i.e., flavonols, flavones, flavanones, flavan-3-ols, and anthocyanins) and CRC risk in the NHS and HPFS, with longer follow-up and a larger number of incident cases compared with the previous analysis (13), thus enabling us to investigate long-term habitual flavonoid intake as well as flavonoid intake with different latencies to better understand whether timing of intake makes a difference.

## METHODS

### Study population

Details of the HPFS and NHS prospective cohorts have been published elsewhere (19, 20). In brief, the HPFS was initiated in 1986 and included 51,529 US male health professionals (dentists, optometrists, osteopaths, podiatrists, pharmacists, veterinarians) who were 40–75 y old at baseline. The NHS began in 1976, when 121,701 US-registered female nurses aged 30–55 y provided information on their medical history and lifestyle. In both cohorts, questionnaires were administered biennially to update lifestyle factors and elicit new medical diagnoses. Follow-up rates have been >90% in each 2-y cycle, and the cumulative follow-up rate (i.e., the percentage of potentially collected person-years) was 94% in the HPFS and 93% in the NHS. Dietary intake data were collected every 4 y in both the NHS and HPFS via a mailed food-frequency questionnaire (FFQ).

In the present study, we used 1984 as baseline for the NHS and 1986 for the HPFS, the respective years in which dietary intakes were assessed by using a validated 116- to 131-item FFQ. Among the participants who returned the baseline FFQs, we excluded those who had a history of cancer (except for nonmelanoma skin cancer) or ulcerative colitis, reported very high or very low energy intakes (<600 or >3500 kcal/d for women, <800 or >4200 kcal/d for men), left >70 food items blank on the FFQs, or had missing information on flavonoid intake. After these exclusions, 76,364 women and 42,478 men were included in the analysis.

The study protocol was approved by the Institutional Review Board at Brigham and Women’s Hospital and the Harvard School of Public Health.

### Assessment of flavonoid intake

Validated FFQs (20, 21) were administered in 1984, 1986, and every 4 y thereafter in the NHS. In HPFS, similar FFQs were administered in 1986 and every 4 y thereafter. In the semiquantitative FFQs, food and beverage items are specified by commonly used units or portion sizes. In each FFQ, participants were asked to report how often, on average, they consumed a serving of each food item during the previous year with 9 possible response categories ranging from “never or less than once per month” to “6 or more times per day.” Individual nutrient intakes were calculated from FFQ data by multiplying the nutrient content per serving of each food by the indicated frequency of consumption (servings of that food per day) and summing over all foods. A database for the calculation of habitual intake of different flavonoid subclasses was developed as has been described previously (18). For the present analysis, 5 main flavonoid subclasses (expressed as aglycones) commonly consumed in the US diet were considered: flavonols (quercetin, kaempferol, myricetin, isorhamnetin), flavones (apigenin, luteolin), flavanones (eriodictyol, hesperetin, naringenin), flavan-3-ol monomers (catechin, epicatechin, epigallocatechin, epicatechin-3-gallate, epigallocatechin-3-gallate), and anthocyanins (cyanidin, delphinidin, malvidin, pelargonidin, petunidin, peonidin). Total flavonoid intakes were derived by the sum of the components flavonols, flavones, flavanones, flavan-3-ols, anthocyanins, and polymeric/oligomeric flavonoids (including proanthocyanidins, theaflavins, and thearubigins). As has been described previously, the main food sources of flavonols are tea and onions, those of flavones and flavanones are oranges, those of flavan-3ols are tea, those of anthocyanins are blueberries, and those of oligomeric and polymeric flavonoids are tea, apples, and strawberries (18, 22). Nutrient intakes were energy adjusted by the residual method (23). To represent long-term dietary intake, we calculated cumulative average intakes for a given questionnaire cycle by averaging the intake for the current and preceding FFQs (24). Because FFQs administered before 1990 contained fewer questions on some flavonoid-rich fruits and vegetables (e.g., onions were not listed in questionnaires before 1990), in sensitivity analyses, we used 1990 as baseline for both the HPFS and NHS.

Reproducibility and relative validity of the FFQs have been investigated previously (25, 26). For the major food sources of flavonoids, correlations between dietary records and FFQs in the HPFS and NHS were as follows: 0.77–0.93 for tea, 0.83–0.90 for red wine, 0.74–0.76 for oranges, and 0.70–0.80 for apples or pears (25, 26). In recent analyses in the HPFS and NHS, flavonoid subclasses have been associated with lower risk of several chronic diseases, including hypertension (18), ovarian cancer (27), and Parkinson disease (28), giving further qualitative support for the ability of the FFQ to reasonably estimate dietary intake of flavonoid subclasses.

### Assessment of covariables

Information on lifestyle and other health-related factors, such as body weight, cigarette smoking, physical activity, participation in gastrointestinal endoscopic examinations, use of aspirin and other nonsteroidal anti-inflammatory drugs, menopausal status, and postmenopausal hormone use (in women only), was collected and updated in the biennial follow-up questionnaires. Family history of CRC in a first-degree relative was assessed in the 1982, 1988, 1992, 1996, and 2000 questionnaires in the NHS and in the 1986, 1990, 1992, and 1996 questionnaires in the HPFS.

### Ascertainment of CRC cases

In both the NHS and HPFS, participants who reported a diagnosis of CRC on one of the biennial follow-up questionnaires were asked for permission to obtain their medical records and pathologic reports. Given the colorectal continuum model (29), we included colon cancers and rectal cancers. A study physician, blinded to exposure information, reviewed the records to confirm CRC diagnosis and to extract information on anatomic location, stage, and histologic type of the cancer. The National Death Index was queried to discover deaths and to ascertain any diagnosis of CRC that contributed to death or was a secondary diagnosis. In the case participants who had died of CRC were not captured during our regular disease follow-up, we contacted next of kin to ask for permission to obtain and review medical records to confirm a diagnosis of CRC. Deaths of all cohort members were identified through the National Death Index, the state vital statistics record, and death certificates that were mailed by next of kin of deceased participants, resulting in >98% complete ascertainment in mortality surveillance (30, 31). CRC was defined based on the International Statistical Classification of Diseases, Injury and Causes of Death (Ninth Revision) as a combination of tumors of the colon (C18.0–C18.7), tumors that were overlapping or unspecified (C18.8–C18.9), and tumors of the rectum (C19–C20). All cases included in the present analysis were confirmed by medical records. Between return of the baseline questionnaires and 2010, a total of 1458 CRC cases (1151 colon, 307 rectum) were documented in the NHS and 1061 (831 colon, 230 rectum) in the HPFS.

### Statistical analysis

Each participant contributed person-time of follow-up from the date of return of the baseline questionnaire to the date of CRC diagnosis, death, or end of analysis follow-up (31 May 2010 for the NHS, 31 January 2010 for the HPFS), whichever came first. We used Cox proportional hazards regression models calculating HRs (as an estimate of RR) and 95% CIs to quantify the associations of habitual intake of different flavonoid subclasses with overall and subsite-specific risk of CRC within each cohort. To control as finely as possible for confounding by age and calendar time, we stratified Cox models jointly by age in months at the start of follow-up and calendar year of the current questionnaire cycle. We tested the proportional hazards assumption by using likelihood ratio tests comparing models with and without interaction terms between flavonoid subclass variables and age or follow-up time and did not observe any violation. In multivariable models, on the basis of our knowledge from previous investigations within the HPFS and NHS (32–34), we simultaneously adjusted for CRC risk factors that may confound the association between flavonoid subclasses and CRC risk. These factors included pack-years of smoking before age 30 y, history of CRC in a parent or sibling, history of lower bowel endoscopy, regular aspirin use, menopausal hormone use (in women only), BMI, physical activity, alcohol consumption, total calorie intake, vitamin D intake, calcium intake, and consumption of red meat and processed meat. To assess whether potential collinearity between dietary variables may have affected our results, we also constructed a “basic model” (35), which included all nondietary covariates, alcohol, and total energy intake and then added each dietary variable separately to the model. To represent long-term dietary and lifestyle patterns, we used cumulative averages for BMI, physical activity, regular aspirin/nonsteroidal anti-inflammatory drug use, and dietary intakes throughout follow-up to control for confounding in regression models. As a sensitivity analysis, we also adjusted for the diet quality scores from the Alternate Healthy Eating Index–2010 (36) and Dietary Approaches to Stop Hypertension (37). Our primary exposure of interest was cumulative average flavonoid intake between baseline and follow-up period representing long-term habitual flavonoid intake and minimizing the impact of random measurement errors in the dietary assessment by FFQs.

In sensitivity analyses, we evaluated the possible modification by time in the association between intake of flavonoid subclasses and CRC incidence. For this purpose, Cox models based on dietary data collected at different time points were constructed: *1*) flavonoid intake at the beginning of follow-up, *2*) recent (simple updated) flavonoid intake reported on the most recent FFQ before each follow-up interval (latency 0–4 y), and *3*) flavonoid intake reported at different latencies (i.e., 4–8 y, 8–12 y, 12–16 y) before diagnosis as described previously (38). For example, for latency of 4–8 y, flavonoid intake assessed on the 1986 FFQ was related to CRC cases diagnosed between 1990 and 1994, flavonoid intake assessed on the 1990 FFQ was related to CRC cases diagnosed between 1994 and 1998, flavonoid intake assessed on the 1998 FFQ was related to CRC cases diagnosed between 2002 and 2006, and so forth. Because the FFQs were administered every 4 y, the simple updated model 2 can be considered a 0- to 4-y latency analysis.

Intakes of flavonoid subclasses were categorized into quintiles based on the distribution in the study population. Tests for linear trend over quintiles were performed by assigning the median value to each category, entering these values as a continuous variable to the model, and evaluating its statistical significance by using the Wald test. All analyses were conducted in the NHS and HPFS separately, and risk estimates were pooled by using random-effects models (39) if there was no indication of heterogeneity by sex. We tested whether the association between flavonoid subclass intake and risk of CRC was modified by age (<65 y vs. ≥65 y), smoking status (ever vs. never), alcohol intake (high vs. low, based on sex-specific median value), or physical activity (high vs. low, based on sex-specific median value) by adding cross-product terms to the multivariable models and employing Wald tests.

All analyses were conducted by using SAS software (version 9.3; SAS Institute Inc.). A 2-sided *P* value of <0.05 was considered statistically significant.

## RESULTS

Participants’ characteristics, averaged according to proportion of person-time in each quintile of total flavonoid intake, are shown in **Table 1** (equivalent tables for intake of flavonoid subclasses are available as **Supplemental Tables 1–5**). In both men and women, physical activity was higher in participants with high flavonoid intake than in participants in the lower quintiles of flavonoid intake. Dietary intakes of red meat and processed meat were lower in the upper quintiles of flavonoid intake than in the lower quintiles. In the HPFS, during 419,133 y of follow-up, 1061 cases of CRC occurred (2.53 cases per 1000 person-years). In the NHS, 1458 CRC cases were registered during 927,994 person-years of follow-up (1.57 cases per 1000 person-years).

**TABLE 1:** Age-standardized characteristics of person-years according to quintiles of total flavonoid intake in the NHS and HPFS^1^

	HPFS			NHS		
	Q1	Q3	Q5	Q1	Q3	Q5
Age at baseline, y	51.4 ± 9.3^2^	52.4 ± 9.4	52.9 ± 9.3	49.3 ± 7.1	50.4 ± 7.1	50.1 ± 7.2
BMI, kg/m^2^	26.3 ± 3.8	25.8 ± 3.5	25.7 ± 3.5	26.6 ± 5.5	26.1 ± 5.1	26.0 ± 5.0
Physical activity, METs/wk	28.3 ± 35.8	35.5 ± 40.5	34.8 ± 40.2	13.6 ± 19.3	18.2 ± 22.6	18.0 ± 23.1
Current/past smokers, %	10.1	4.1	4.1	22.1	10.9	11.2
Pack-years of smoking before age 30 y	11.7 ± 6.7	10.7 ± 6.5	10.8 ± 6.6	7.5 ± 5.3	6.8 ± 5.4	6.9 ± 5.5
Family history of CRC, %	11.0	11.8	11.7	12.9	13.3	13.4
History of endoscopy, %	19.0	21.0	21.2	18.7	20.1	19.9
Regular aspirin or NSAID use, %	39.8	40.7	40.6	41.6	41.0	42.0
Dietary intake						
Alcohol, g/d	12.1 ± 16.8	11.0 ± 14.2	9.9 ± 13.9	6.5 ± 12.0	5.5 ± 9.3	4.8 ± 9.2
Calcium, mg/d	952 ± 470	973 ± 446	968 ± 475	1097 ± 595	1197 ± 586	1184 ± 610
Vitamin D, IU/d	423 ± 304	451 ± 301	461 ± 326	395 ± 303	431 ± 301	433 ± 313
Red meat, g/d	74.1 ± 55.3	59.2 ± 45.6	53.0 ± 46.0	61.9 ± 44.0	53.3 ± 38.5	50.8 ± 38.9
Processed meat, g/d	13.4 ± 16.6	10.1 ± 12.4	8.4 ± 11.8	10.1 ± 12.3	7.8 ± 9.6	7.2 ± 9.5
Total flavonoids, mg/d	116 ± 36	267 ± 33	769 ± 355	107 ± 62	251 ± 62	808 ± 390
Flavonols, mg/d	10.5 ± 6.5	16.3 ± 8.2	32.0 ± 14.3	9.1 ± 5.8	14.6 ± 7.2	31.9 ± 13.6
Flavones, mg/d	1.5 ± 1.3	2.9 ± 1.6	3.4 ± 2.4	1.3 ± 1.1	2.4 ± 1.5	2.5 ± 2.0
Flavanones, mg/d	25.9 ± 23.3	57.9 ± 38.3	68.9 ± 59.7	21.4 ± 21.2	48.7 ± 35.8	49.8 ± 45.5
Flavan-3-ols, mg/d	11.9 ± 7.2	26.5 ± 10.8	128 ± 88	9.3 ± 5.1	24.1 ± 10.3	149.5 ± 95.1
Anthocyanins, mg/d	5.5 ± 5.4	14.5 ± 11.1	25.5 ± 36.1	5.5 ± 5.4	14.6 ± 11.3	22.5 ± 31.6
Polymers, mg/d	60.4 ± 26.3	149 ± 39	517 ± 279	54.1 ± 23.6	141.2 ± 35.5	565.8 ± 301.6

1 All variables (except age) are age standardized. Physical activity is described by the product sum of the MET of each specific recreational activity and hours spent on the activity per week. Regular aspirin or NSAID use is defined as ≥2 standard (325-mg) tablets of aspirin or ≥2 tablets of NSAIDs/wk. CRC, colorectal cancer; HPFS, Health Professionals Follow-Up Study; MET, metabolic equivalent; NHS, Nurses’ Health Study; NSAID, nonsteroidal anti-inflammatory drug; Q, quintile.

2 Mean ± SD (all such values).

In relation to the different flavonoid subclasses, flavonol, flavone, flavanone, or flavan-3-ol intake was not associated with risk of CRC in pooled analyses or cohort-specific analyses (**Table 2**). Anthocyanin intake was statistically significantly associated with lower risk of CRC in age-adjusted models in men (RR: 0.78; 95% CI: 0.64, 0.94; *P*-trend = 0.03), which was attenuated after multivariable adjustment (RR: 0.88; 95% CI: 0.72, 1.07; *P*-trend = 0.35). We explored the reason for this attenuation by adding *1*) nondietary potential confounders one by one to the age-adjusted model and *2*) dietary potential confounders one by one to the basic model (for more detail on the basic model, see Statistical analysis section). Largest attenuations were observed after individual adjustment for physical activity, smoking before age 30 y, and red and processed meat intake (data not shown). However, no association between anthocyanin intake and risk of CRC was observed in women, and there was no substantial heterogeneity by sex (*P*-heterogeneity = 0.18).

**TABLE 2:** RRs (95% CIs) of colorectal cancer and subsites according to quintiles of flavonoid intake^1^

	RR (95% CI)						
	Q1	Q2	Q3	Q4	Q5	*P*-trend	*P*-het
Flavonols							
Colorectal							
Men (1061 cases)							
Age adjusted	1 (reference)	0.82 (0.67, 1.01)	0.88 (0.72, 1.07)	1.06 (0.88, 1.28)	0.93 (0.76, 1.12)	0.72	
Multivariable	1 (reference)	0.83 (0.68, 1.02)	0.92 (0.75, 1.13)	1.12 (0.92, 1.36)	1.00 (0.82, 1.22)	0.25	
Women (1458 cases)							
Age adjusted	1 (reference)	0.96 (0.81, 1.13)	0.87 (0.74,1.03)	1.01 (0.86,1.19)	1.00 (0.85, 1.18)	0.57	
Multivariable	1 (reference)	0.99 (0.84,1.17)	0.92 (0.78, 1.09)	1.07 (0.91,1.27)	1.06 (0.90, 1.26)	0.23	
Pooled (2519 cases), multivariable	1(reference)	0.92 (0.78, 1.09)	0.92 (0.81, 1.05)	1.10 (0.97, 1.24)	1.04 (0.91, 1.18)	0.10	0.92
Colon							
Men (831 cases), multivariable	1 (reference)	0.79 (0.62, 1.00)	0.94 (0.75, 1.18)	1.14 (0.92, 1.42)	1.02 (0.82, 1.28)	0.16	
Women (1151 cases), multivariable	1 (reference)	0.95 (0.79, 1.15)	0.86 (0.71, 1.05)	1.02 (0.85, 1.23)	1.01 (0.83, 1.22)	0.54	
Pooled (1982 cases), multivariable	1 (reference)	0.88 (0.73, 1.06)	0.90 (0.77, 1.04)	1.07 (0.93, 1.23)	1.01 (0.86, 1.17)	0.16	0.50
Rectum							
Men (230 cases), multivariable	1 (reference)	0.96 (0.63, 1.47)	0.84 (0.54, 1.30)	1.05 (0.69, 1.61)	0.92 (0.59, 1.42)	0.86	
Women (307 cases), multivariable	1 (reference)	1.13 (0.78, 1.66)	1.18 (0.81, 1.72)	1.31 (0.90, 1.89)	1.30 (0.90, 1.90)	0.15	
Pooled (537 cases), multivariable	1(reference)	1.05 (0.80, 1.40)	1.01 (0.73, 1.41)	1.19 (0.90, 1.57)	1.11 (0.79, 1.57)	0.37	0.29
Flavones							
Colorectal							
Men (1061 cases)							
Age adjusted	1 (reference)	0.96 (0.79, 1.17)	0.95 (0.78, 1.16)	0.91 (0.75, 1.11)	0.90 (0.74, 1.09)	0.26	
Multivariable	1 (reference)	0.99 (0.81, 1.21)	1.03 (0.84, 1.25)	1.02 (0.83, 1.24)	1.04 (0.85, 1.27)	0.67	
Women (1458 cases)							
Age adjusted	1 (reference)	0.94 (0.80, 1.12)	0.94 (0.79, 1.11)	0.90 (0.76, 1.06)	0.90 (0.76, 1.06)	0.17	
Multivariable	1 (reference)	0.99 (0.83, 1.17)	1.01 (0.85, 1.19)	0.98 (0.83, 1.17)	0.99 (0.83, 1.18)	0.91	
Pooled (2519 cases), multivariable	1 (reference)	0.99 (0.87, 1.13)	1.02 (0.89, 1.16)	1.00 (0.88, 1.14)	1.01 (0.89, 1.15)	0.81	0.71
Colon							
Men (831 cases), multivariable	1 (reference)	1.00 (0.80, 1.25)	1.04 (0.83, 1.30)	0.99 (0.79, 1.25)	1.12 (0.89, 1.40)	0.33	
Women (1151 cases), multivariable	1 (reference)	0.97 (0.80, 1.18)	1.01 (0.84, 1.23)	1.00 (0.83, 1.22)	1.00 (0.83, 1.22)	0.66	
Pooled (1982 cases), multivariable	1 (reference)	0.98 (0.85, 1.14)	1.02 (0.88, 1.18)	1.00 (0.86, 1.16)	1.07 (0.92, 1.24)	0.31	0.75
Rectum							
Men (230 cases), multivariable	1 (reference)	0.96 (0.63, 1.45)	0.97 (0.64, 1.49)	1.10 (0.73, 1.67)	0.75 (0.47, 1.19)	0.34	
Women (307 cases), multivariable	1 (reference)	1.05 (0.73, 1.49)	0.99 (0.69, 1.43)	0.92 (0.64, 1.34)	0.85 (0.58, 1.24)	0.27	
Pooled (537 cases), multivariable	1 (reference)	1.01 (0.77, 1.32)	0.99 (0.75, 1.30)	1.00 (0.76, 1.32)	0.81 (0.60, 1.08)	0.15	0.87
Flavanones							
Colorectal							
Men (1061 cases)							
Age adjusted	1 (reference)	1.05 (0.86, 1.28)	1.08 (0.89, 1.31)	0.99 (0.82, 1.21)	0.87 (0.71, 1.06)	0.08	
Multivariable	1 (reference)	1.10 (0.90, 1.34)	1.17 (0.96, 1.43)	1.12 (0.91, 1.36)	1.02 (0.83, 1.25)	0.98	
Women (1458 cases)							
Age adjusted	1 (reference)	0.88 (0.74, 1.04)	0.90 (0.76, 1.06)	0.82 (0.69, 0.96)	0.84 (0.72, 1.00)	0.05	
Multivariable	1 (reference)	0.90 (0.76, 1.07)	0.96 (0.81, 1.13)	0.89 (0.75, 1.05)	0.93 (0.78, 1.10)	0.48	
Pooled (2519 cases), multivariable	1 (reference)	0.99 (0.82, 1.19)	1.05 (0.86, 1.28)	0.99 (0.79, 1.23)	0.96 (0.84, 1.10)	0.62	0.62
Colon							
Men (831 cases), multivariable	1 (reference)	1.07 (0.85, 1.35)	1.18 (0.94, 1.47)	1.16 (0.92, 1.45)	1.01 (0.80, 1.28)	0.90	
Women (1151 cases), multivariable	1 (reference)	0.87 (0.72, 1.05)	0.90 (0.74, 1.08)	0.91 (0.75, 1.09)	0.93 (0.77, 1.13)	0.77	
Pooled (1982 cases), multivariable	1 (reference)	0.96 (0.78, 1.17)	1.02 (0.78, 1.33)	1.02 (0.80, 1.29)	0.96 (0.83, 1.12)	0.92	0.77
Rectum							
Men (230 cases), multivariable	1 (reference)	1.15 (0.75, 1.75)	1.14 (0.75, 1.74)	0.94 (0.61, 1.47)	1.00 (0.64, 1.56)	0.66	
Women (307 cases), multivariable	1 (reference)	1.04 (0.72, 1.49)	1.21 (0.85, 1.72)	0.82 (0.56, 1.21)	0.92 (0.63, 1.34)	0.34	
Pooled (537 cases), multivariable	1 (reference)	1.08 (0.82, 1.42)	1.18 (0.90, 1.54)	0.87 (0.65, 1.17)	0.95 (0.71, 1.27)	0.33	0.69
Flavan-3-ols							
Colorectal							
Men (1061 cases)							
Age adjusted	1 (reference)	0.94 (0.77, 1.15)	0.97 (0.80, 1.18)	1.00 (0.83, 1.21)	1.03 (0.85, 1.25)	0.48	
Multivariable	1 (reference)	0.99 (0.81, 1.21)	1.05 (0.86, 1.28)	1.09 (0.89, 1.33)	1.12 (0.92, 1.36)	0.18	
Women (1458 cases)							
Age adjusted	1 (reference)	0.89 (0.75, 1.05)	0.88 (0.74, 1.03)	0.89 (0.76, 1.05)	0.99 (0.84, 1.16)	0.40	
Multivariable	1 (reference)	0.93 (0.79, 1.11)	0.93 (0.79, 1.10)	0.94 (0.80, 1.11)	1.04 (0.88, 1.23)	0.25	
Pooled (2519 cases), multivariable	1 (reference)	0.96 (0.84, 1.09)	0.98 (0.86, 1.11)	1.00 (0.87, 1.15)	1.07 (0.95, 1.21)	0.09	0.65
Colon							
Men (831 cases), multivariable	1 (reference)	1.07 (0.85, 1.34)	1.12 (0.89, 1.41)	1.16 (0.93, 1.46)	1.20 (0.96, 1.50)	0.13	
Women (1151 cases), multivariable	1 (reference)	0.92 (0.77, 1.11)	0.95 (0.79, 1.14)	0.85 (0.70, 1.02)	1.02 (0.85, 1.23)	0.52	
Pooled (1982 cases), multivariable	1 (reference)	0.98 (0.85, 1.13)	1.02 (0.87, 1.19)	0.99 (0.72, 1.35)	1.09 (0.93, 1.27)	0.16	0.39
Rectum							
Men (230 cases), multivariable	1 (reference)	0.77 (0.51, 1.18)	0.85 (0.56, 1.28)	0.89 (0.59, 1.35)	0.90 (0.60, 1.35)	0.97	
Women (307 cases), multivariable	1 (reference)	0.97 (0.67, 1.42)	0.85 (0.58, 1.25)	1.39 (0.98, 1.98)	1.13 (0.79, 1.63)	0.20	
Pooled (537 cases), multivariable	1 (reference)	0.88 (0.66, 1.16)	0.85 (0.64, 1.13)	1.13 (0.73, 1.75)	1.02 (0.78, 1.34)	0.25	0.50
Anthocyanins							
Colorectal							
Men (1061 cases)							
Age adjusted	1 (reference)	0.83 (0.69, 1.01)	0.92 (0.76, 1.10)	0.84 (0.69, 1.02)	0.78 (0.64, 0.94)	0.03	
Multivariable	1 (reference)	0.88 (0.73, 1.07)	0.99 (0.82, 1.20)	0.94 (0.77, 1.14)	0.88 (0.72, 1.08)	0.36	
Women (1458 cases)							
Age adjusted	1 (reference)	0.92 (0.78, 1.09)	1.01 (0.86, 1.19)	0.91 (0.77, 1.08)	0.96 (0.81, 1.13)	0.71	
Multivariable	1 (reference)	0.96 (0.81, 1.14)	1.08 (0.92, 1.28)	0.99 (0.84, 1.18)	1.08 (0.90, 1.28)	0.35	
Pooled (2519 cases), multivariable	1 (reference)	0.93 (0.82, 1.05)	1.04 (0.92, 1.18)	0.97 (0.85, 1.10)	0.98 (0.81, 1.19)	0.98	0.19
Colon							
Men (831 cases), multivariable	1 (reference)	0.92 (0.74, 1.14)	0.98 (0.79, 1.21)	1.00 (0.81, 1.25)	0.85 (0.67, 1.07)	0.26	
Women (1151 cases), multivariable	1 (reference)	0.96 (0.79, 1.15)	1.06 (0.88, 1.28)	0.98 (0.81, 1.19)	1.04 (0.86, 1.27)	0.57	
Pooled (1982 cases), multivariable	1 (reference)	0.94 (0.81, 1.08)	1.02 (0.89, 1.18)	0.99 (0.86, 1.15)	0.95 (0.77, 1.16)	0.76	0.23
Rectum							
Men (230 cases), multivariable	1 (reference)	0.74 (0.48, 1.13)	1.01 (0.67, 1.50)	0.70 (0.45, 1.10)	0.99 (0.65, 1.50)	0.83	
Women (307 cases), multivariable	1 (reference)	1.00 (0.69, 1.45)	1.17 (0.81, 1.68)	1.04 (0.71, 1.52)	1.04 (0.71, 1.52)	0.35	
Pooled (537 cases), multivariable	1 (reference)	0.88 (0.66, 1.17)	1.09 (0.83, 1.43)	0.87 (0.60, 1.27)	1.10 (0.83, 1.45)	0.40	0.63

1 Multivariable relative risks were adjusted for age (in months), pack-years of smoking before age 30 y (0, 1–4, 5–10, or >10 pack-years), history of colorectal cancer in a parent or sibling (yes or no), history of endoscopy (yes or no), regular aspirin use (yes or no), BMI (in kg/m^2^; <25, 25 to <30, ≥30), physical activity (low, medium, high), alcohol consumption (0 to <5, 5 to <10, 10 to <15, or ≥15 g/d), total calories (quintiles), energy-adjusted total vitamin D intake (quintiles), total calcium intake (quintiles), red meat intake (quintiles), and processed meat intake (quintiles). *P*-het, *P* value for heterogeneity by sex; Q, quintile.

When examining subsites of CRC, no association between flavonoid subclasses and risk of colon cancer (neither proximal nor distal colon cancer; data not shown) or rectal cancer was observed in both cohorts.

The associations were not materially altered after additional adjustment for Alternate Healthy Eating Index–2010 score or Dietary Approaches to Stop Hypertension diet scores (data not shown).

When we investigated potential effect modification in the association between flavonoid subclass intake and risk of CRC by age, smoking, alcohol intake, and physical activity, no statistically significant interactions were observed (all *P*-interaction > 0.05).

Associations between flavonoid subclasses and risk of CRC were not modified by time—that is, no substantially different results were obtained when flavonoid intake at beginning of follow-up (**Supplemental Table 6**) or with a latency of 0–4 y, 4–8 y, or 8–12 y (**Supplemental Table 7**) was investigated. The association between intake of flavonols, of which onions are a main food source, and risk of CRC was not substantially different when follow-up was started in 1990 (the first year onions were specifically asked for in the FFQs) (data not shown).

We further explored the association between main food sources of flavonoids and risk of colorectal, colon, and rectal cancer (**Supplemental Table 8**). Dietary intake of blueberries (main food source of anthocyanins), oranges (main food source of flavones and flavanones), or tea (main food source of flavonols and flavan-3-ols) was not associated with risk of CRC in men or women.

## DISCUSSION

In this study comprising 2 large cohorts with >1,347,000 person-years of follow-up and 2519 CRC cases, we observed no association between habitual intake of any flavonoid subclass in relation to risk of CRC after multivariable adjustment. Thus, these findings do not support the hypothesis that dietary flavonoid intake is associated with a lower risk of CRC. To our knowledge, this is the largest prospective study relating habitual intake of various subclasses of flavonoids to risk of CRC.

Epidemiologic data investigating flavonoid intake in relation to CRC risk are conflicting. Inverse associations between flavonols, flavan-3-ols, and anthocyanins and risk of CRC have been observed in case-control studies (40). However, because flavonoids originate largely from plant-based foods and beverages (i.e., foods generally considered healthy), differential recall of these foods by CRC cases compared with healthy control participants is a likely source of bias in these studies. Our findings do not support the findings from the secondary analysis of the Polyp Prevention Trial, in which flavonol intake was associated with a lower risk of advanced adenoma recurrence (16). If flavonols truly influence the development of adenomas, we would expect that early flavonol intake should have a stronger association with risk of CRC than recent intake. However, the latency analysis gave only weak support for flavonol intake 12–16 y before diagnosis being inversely associated with risk of CRC. Our findings also do not confirm the previously observed associations between flavan-3-ol intake and lower risk of rectal cancer (15) or urinary flavan-3-ols and lower risk of colon cancer (17), but they are in line with a meta-analysis including 3 prospective studies in which no statistically significant association between flavan-3-ol intake and risk of CRC was observed (40). Furthermore, the lack of association between dietary intake of flavonols, flavones (10, 12, 14), and flavanones (41, 42) and risk of CRC observed here is in line with previous prospective studies.

Our results showed no clear association between anthocyanin intake and risk of CRC. In 2 previous prospective studies (41, 42), no associations between anthocyanins and risk of CRC were observed. It has been suggested that colonic microbial catabolites of anthocyanins may mediate biological activities, including anticarcinogenic properties (4). Because the catabolic yields differ largely among individuals depending on the composition of the gut microflora, it is possible that in an epidemiologic study such as the present one, a reduced risk of CRC could have been observed only in individuals with a certain microflora. The growing research on the gut microbiome may enable future epidemiologic studies to take this into account.

Of the major flavonoid food sources, tea, oranges, and blueberries were not associated with risk of CRC in our study. This is in line with a recently published meta-analysis on tea (43) intake in relation to risk of CRC and previous prospective studies investigating citrus fruit intake and risk of CRC (44, 45). We are not aware of any prior prospective study investigating blueberry intake in relation to CRC.

Although our study and the majority of prospective studies do not support an inverse association between dietary intake of flavonoid subclasses and risk of CRC, the evidence from experimental studies remains biologically plausible. In in vitro studies, flavonoids, especially flavan-3-ols and flavonols from tea, have been shown to inhibit growth of human colon cancer cell lines (46). In addition, it has been shown that flavan-3-ols from apples can reactivate silenced tumor suppressor genes in CRC cells (47). However, the evidence on protective effects of flavonoids against colon carcinogenesis is less conclusive in animal studies (47). It remains unclear whether anticarcinogenic effects of flavonoid subclasses observed in experimental studies play a relevant role in the etiology of CRC in humans because the flavonoid concentrations used in in vitro studies or doses used in in vivo studies can hardly be reached through habitual dietary intake (3). For example, studies investigating the effect of dietary intake of the flavonol quercetin on tumor formation in mice typically used a quercetin concentration of 2% in the diet (48). Also, many human intervention studies investigating the effect of flavonoid intake on intermediary markers such as antioxidant biomarkers or carcinogenesis markers such as DNA damage used experimental doses that are difficult to attain with habitual diet (e.g., daily doses of 50–500 mg quercetin) (49). For comparison, median intakes of flavonols were 18 mg/d in the HPFS and 15 mg/d in the NHS. Bioavailability studies have shown that a 50-mg dose of quercetin leads to plasma concentrations of 0.75–1.5 μmol/L quercetin (49), which is 10-fold lower than the typical dose of 10 μM used in in vitro studies with colon cancer cell lines (2). However, because a substantial proportion of dietary flavonoids reaches the colon (4), it is expected that at least some of the biological activity, including potential anticarcinogenic properties, may act locally through colonic metabolites, but these effects are difficult to quantify.

Our study has several limitations. First, the FFQ was not specifically designed to investigate flavonoid intakes. Therefore, some foods rich in flavonoids (e.g., herbs) were missing from the FFQ, and foods with varying flavonoid contents may have been subsumed within one food item (e.g., apples and pears). Furthermore, flavonoid contents may vary depending on species, seasonal variation, maturity, and various agricultural, food-processing, and storage practices. These factors may have contributed to misclassification of flavonoid intakes, which may have led to an attenuation of observed associations, and thus may have obscured the detection of weak associations. Another limitation lies in the multiple comparisons that were made during this hypothesis-driven data analysis. We also acknowledge that our findings are not necessarily generalizable to other populations. Strengths of our study include the prospective study design; the repeated dietary intake assessment, which allowed for estimation of long-term intake as well as investigation of different latencies; and the large number of cases, which enabled us to investigate CRC individually according to location. Furthermore, the detailed data on potentially confounding factors are a strength and turned out to be important, because some of the associations that were observed in age-adjusted models were largely attenuated in the fully adjusted models.

In conclusion, our study findings do not support the hypothesis that diets high in flavonoids may reduce risk of CRC. The lack of association may be related to the low habitual intake of flavonoids, interindividual variation of colonic microbial catabolite production, and/or misclassification of flavonoid intake. Epidemiologic studies employing biomarkers of flavonoids may help elucidate whether the anticarcinogenic effects of flavonoids that have been observed in experimental studies may also be relevant in humans with habitual dietary intake.

## Footnotes

12 Abbreviations used: CRC, colorectal cancer; FFQ, food-frequency questionnaire; HPFS, Health Professionals Follow-Up Study; NHS, Nurses’ Health Study.

## References

[1] Moon YJ, Wang X, Morris ME Dietary flavonoids: effects on xenobiotic and carcinogen metabolism. Toxicol In Vitro 2006;20:187–210. 16289744 10.1016/j.tiv.2005.06.048

[2] Kandaswami C, Lee LT, Lee PP, Hwang JJ, Ke FC, Huang YT, Lee MT The antitumor activities of flavonoids. In Vivo 2005;19:895–909. Corrected and republished from: In Vivo 2007;21:553. 16097445

[3] Yang CS, Landau JM, Huang MT, Newmark HL Inhibition of carcinogenesis by dietary polyphenolic compounds. Annu Rev Nutr 2001;21:381–406. 11375442 10.1146/annurev.nutr.21.1.381

[4] Williamson G, Clifford MN Colonic metabolites of berry polyphenols: the missing link to biological activity? Br J Nutr 2010;104(Suppl 3):S48–66. 20955650 10.1017/S0007114510003946

[5] Rossi M, Negri E, Talamini R, Bosetti C, Parpinel M, Gnagnarella P, Franceschi S, Dal Maso L, Montella M, Giacosa A, Flavonoids and colorectal cancer in Italy. Cancer Epidemiol Biomarkers Prev 2006;15:1555–8. 16896049 10.1158/1055-9965.EPI-06-0017

[6] Theodoratou E, Kyle J, Cetnarskyj R, Farrington SM, Tenesa A, Barnetson R, Porteous M, Dunlop M, Campbell H Dietary flavonoids and the risk of colorectal cancer. Cancer Epidemiol Biomarkers Prev 2007;16:684–93. 17416758 10.1158/1055-9965.EPI-06-0785

[7] Rossi M, Bosetti C, Negri E, Lagiou P, La Vecchia C Flavonoids, proanthocyanidins, and cancer risk: a network of case-control studies from Italy. Nutr Cancer 2010;62:871–7. 20924962 10.1080/01635581.2010.509534

[8] Zamora-Ros R, Not C, Guino E, Lujan-Barroso L, Garcia RM, Biondo S, Salazar R, Moreno V Association between habitual dietary flavonoid and lignan intake and colorectal cancer in a Spanish case-control study (the Bellvitge Colorectal Cancer Study). Cancer Causes Control 2013;24:549–57. 22588680 10.1007/s10552-012-9992-z

[9] Kyle JA, Sharp L, Little J, Duthie GG, McNeill G Dietary flavonoid intake and colorectal cancer: a case-control study. Br J Nutr 2010;103:429–36. 19732470 10.1017/S0007114509991784

[10] Hirvonen T, Virtamo J, Korhonen P, Albanes D, Pietinen P Flavonol and flavone intake and the risk of cancer in male smokers (Finland). Cancer Causes Control 2001;12:789–96. 11714106 10.1023/a:1012232008016

[11] Knekt P, Jarvinen R, Seppanen R, Hellovaara M, Teppo L, Pukkala E, Aromaa A Dietary flavonoids and the risk of lung cancer and other malignant neoplasms. Am J Epidemiol 1997;146:223–30. 9247006 10.1093/oxfordjournals.aje.a009257

[12] Knekt P, Kumpulainen J, Jarvinen R, Rissanen H, Heliovaara M, Reunanen A, Hakulinen T, Aromaa A Flavonoid intake and risk of chronic diseases. Am J Clin Nutr 2002;76:560–8. 12198000 10.1093/ajcn/76.3.560

[13] Lin J, Zhang SM, Wu K, Willett WC, Fuchs CS, Giovannucci E Flavonoid intake and colorectal cancer risk in men and women. Am J Epidemiol 2006;164:644–51. 16923774 10.1093/aje/kwj296

[14] Simons CC, Hughes LA, Arts IC, Goldbohm RA, van den Brandt PA, Weijenberg MP Dietary flavonol, flavone and catechin intake and risk of colorectal cancer in the Netherlands Cohort Study. Int J Cancer 2009;125:2945–52. 19530252 10.1002/ijc.24645

[15] Arts IC, Jacobs DR Jr, Gross M, Harnack LJ, Folsom AR Dietary catechins and cancer incidence among postmenopausal women: the Iowa Women’s Health Study (United States). Cancer Causes Control 2002;13:373–82. 12074507 10.1023/a:1015290131096

[16] Bobe G, Sansbury LB, Albert PS, Cross AJ, Kahle L, Ashby J, Slattery ML, Caan B, Paskett E, Iber F, Dietary flavonoids and colorectal adenoma recurrence in the Polyp Prevention Trial. Cancer Epidemiol Biomarkers Prev 2008;17:1344–53. 18559549 10.1158/1055-9965.EPI-07-0747 PMC2517243

[17] Yuan JM, Gao YT, Yang CS, Yu MC Urinary biomarkers of tea polyphenols and risk of colorectal cancer in the Shanghai Cohort Study. Int J Cancer 2007;120:1344–50. 17149697 10.1002/ijc.22460

[18] Cassidy A, O’Reilly EJ, Kay C, Sampson L, Franz M, Forman JP, Curhan G, Rimm EB Habitual intake of flavonoid subclasses and incident hypertension in adults. Am J Clin Nutr 2011;93:338–47. 21106916 10.3945/ajcn.110.006783 PMC3021426

[19] Rimm EB, Giovannucci EL, Willett WC, Colditz GA, Ascherio A, Rosner B, Stampfer MJ Prospective study of alcohol consumption and risk of coronary disease in men. Lancet 1991;338:464–8. 1678444 10.1016/0140-6736(91)90542-w

[20] Colditz GA, Manson JE, Hankinson SE The Nurses’ Health Study: 20-year contribution to the understanding of health among women. J Womens Health 1997;6:49–62. 9065374 10.1089/jwh.1997.6.49

[21] Willett W Nutritional epidemiology. New York: Oxford University Press; 1998.

[22] Devore EE, Kang JH, Breteler MM, Grodstein F Dietary intakes of berries and flavonoids in relation to cognitive decline. Ann Neurol 2012;72:135–43. 22535616 10.1002/ana.23594 PMC3582325

[23] Willett W, Stampfer MJ Total energy intake: implications for epidemiologic analyses. Am J Epidemiol 1986;124:17–27. 3521261 10.1093/oxfordjournals.aje.a114366

[24] Hu FB, Stampfer MJ, Rimm E, Ascherio A, Rosner BA, Spiegelman D, Willett WC Dietary fat and coronary heart disease: a comparison of approaches for adjusting for total energy intake and modeling repeated dietary measurements. Am J Epidemiol 1999;149:531–40. 10084242 10.1093/oxfordjournals.aje.a009849

[25] Feskanich D, Rimm EB, Giovannucci EL, Colditz GA, Stampfer MJ, Litin LB, Willett WC Reproducibility and validity of food intake measurements from a semiquantitative food frequency questionnaire. J Am Diet Assoc 1993;93:790–6. 8320406 10.1016/0002-8223(93)91754-e

[26] Salvini S, Hunter DJ, Sampson L, Stampfer MJ, Colditz GA, Rosner B, Willett WC Food-based validation of a dietary questionnaire: the effects of week-to-week variation in food consumption. Int J Epidemiol 1989;18:858–67. 2621022 10.1093/ije/18.4.858

[27] Cassidy A, Huang T, Rice MS, Rimm EB, Tworoger SS Intake of dietary flavonoids and risk of epithelial ovarian cancer. Am J Clin Nutr 2014;100:1344–51. 25332332 10.3945/ajcn.114.088708 PMC4196485

[28] Gao X, Cassidy A, Schwarzschild MA, Rimm EB, Ascherio A Habitual intake of dietary flavonoids and risk of Parkinson disease. Neurology 2012;78:1138–45. 22491871 10.1212/WNL.0b013e31824f7fc4 PMC3320056

[29] Yamauchi M, Lochhead P, Morikawa T, Huttenhower C, Chan AT, Giovannucci E, Fuchs C, Ogino S Colorectal cancer: a tale of two sides or a continuum? Gut 2012;61:794–7. 22490520 10.1136/gutjnl-2012-302014 PMC3345045

[30] Stampfer MJ, Willett WC, Speizer FE, Dysert DC, Lipnick R, Rosner B, Hennekens CH Test of the National Death Index. Am J Epidemiol 1984;119:837–9. 6720679 10.1093/oxfordjournals.aje.a113804

[31] Rich-Edwards JW, Corsano KA, Stampfer MJ Test of the National Death Index and Equifax Nationwide Death Search. Am J Epidemiol 1994;140:1016–9. 7985649 10.1093/oxfordjournals.aje.a117191

[32] Platz EA, Willett WC, Colditz GA, Rimm EB, Spiegelman D, Giovannucci E Proportion of colon cancer risk that might be preventable in a cohort of middle-aged US men. Cancer Causes Control 2000;11:579–88. 10977102 10.1023/a:1008999232442

[33] Wei EK, Colditz GA, Giovannucci EL, Fuchs CS, Rosner BA Cumulative risk of colon cancer up to age 70 years by risk factor status using data from the Nurses’ Health Study. Am J Epidemiol 2009;170:863–72. 19723749 10.1093/aje/kwp210 PMC2800259

[34] Giovannucci E Vitamin D and cancer incidence in the Harvard cohorts. Ann Epidemiol 2009;19:84–8. 18291673 10.1016/j.annepidem.2007.12.002

[35] Willett W Issues in analysis and presentation of dietary data. In: Nutritional epidemiology. New York: Oxford University Press; 1998. p. 321–46.

[36] McCullough ML, Feskanich D, Stampfer MJ, Giovannucci EL, Rimm EB, Hu FB, Spiegelman D, Hunter DJ, Colditz GA, Willett WC Diet quality and major chronic disease risk in men and women: moving toward improved dietary guidance. Am J Clin Nutr 2002;76:1261–71. 12450892 10.1093/ajcn/76.6.1261

[37] Sacks FM, Svetkey LP, Vollmer WM, Appel LJ, Bray GA, Harsha D, Obarzanek E, Conlin PR, Miller ER 3rd, Simons-Morton DG, Effects on blood pressure of reduced dietary sodium and the Dietary Approaches to Stop Hypertension (DASH) diet. DASH-Sodium Collaborative Research Group. N Engl J Med 2001;344:3–10. 11136953 10.1056/NEJM200101043440101

[38] Lee JE, Willett WC, Fuchs CS, Smith-Warner SA, Wu K, Ma J, Giovannucci E Folate intake and risk of colorectal cancer and adenoma: modification by time. Am J Clin Nutr 2011;93:817–25. 21270374 10.3945/ajcn.110.007781 PMC3057549

[39] DerSimonian R, Laird N Meta-analysis in clinical trials. Control Clin Trials 1986;7:177–88. 3802833 10.1016/0197-2456(86)90046-2

[40] Woo HD, Kim J Dietary flavonoid intake and risk of stomach and colorectal cancer. World J Gastroenterol 2013;19:1011–9. 23467443 10.3748/wjg.v19.i7.1011 PMC3581988

[41] Cutler GJ, Nettleton JA, Ross JA, Harnack LJ, Jacobs DR Jr, Scrafford CG, Barraj LM, Mink PJ, Robien K Dietary flavonoid intake and risk of cancer in postmenopausal women: the Iowa Women’s Health Study. Int J Cancer 2008;123:664–71. 18491403 10.1002/ijc.23564 PMC2572165

[42] Mursu J, Nurmi T, Tuomainen TP, Salonen JT, Pukkala E, Voutilainen S Intake of flavonoids and risk of cancer in Finnish men: the Kuopio Ischaemic Heart Disease Risk Factor Study. Int J Cancer 2008;123:660–3. 18338754 10.1002/ijc.23421

[43] Yu F, Jin Z, Jiang H, Xiang C, Tang J, Li T, He J Tea consumption and the risk of five major cancers: a dose-response meta-analysis of prospective studies. BMC Cancer 2014;14:197. 24636229 10.1186/1471-2407-14-197 PMC4004325

[44] Park Y, Subar AF, Kipnis V, Thompson FE, Mouw T, Hollenbeck A, Leitzmann MF, Schatzkin A Fruit and vegetable intakes and risk of colorectal cancer in the NIH-AARP diet and health study. Am J Epidemiol 2007;166:170–80. 17485731 10.1093/aje/kwm067

[45] Voorrips LE, Goldbohm RA, van Poppel G, Sturmans F, Hermus RJ, van den Brandt PA Vegetable and fruit consumption and risks of colon and rectal cancer in a prospective cohort study: the Netherlands Cohort Study on Diet and Cancer. Am J Epidemiol 2000;152:1081–92. 11117618 10.1093/aje/152.11.1081

[46] Lambert JD, Hong J, Yang GY, Liao J, Yang CS Inhibition of carcinogenesis by polyphenols: evidence from laboratory investigations. Am J Clin Nutr 2005;81(Suppl):284S–91S. 15640492 10.1093/ajcn/81.1.284S

[47] Fini L, Selgrad M, Fogliano V, Graziani G, Romano M, Hotchkiss E, Daoud YA, De Vol EB, Boland CR, Ricciardiello L Annurca apple polyphenols have potent demethylating activity and can reactivate silenced tumor suppressor genes in colorectal cancer cells. J Nutr 2007;137:2622–8. 18029474 10.1093/jn/137.12.2622

[48] Mahmoud NN, Carothers AM, Grunberger D, Bilinski RT, Churchill MR, Martucci C, Newmark HL, Bertagnolli MM Plant phenolics decrease intestinal tumors in an animal model of familial adenomatous polyposis. Carcinogenesis 2000;21:921–7. 10783313 10.1093/carcin/21.5.921

[49] Williamson G, Manach C Bioavailability and bioefficacy of polyphenols in humans: II. Review of 93 intervention studies. Am J Clin Nutr 2005;81(Suppl):243S–55S. 15640487 10.1093/ajcn/81.1.243S

